# Pediatric Head & Neck Free-Flap Reconstruction Outcomes: Score-Based Effectiveness Assessment

**DOI:** 10.3390/jcm15031091

**Published:** 2026-01-30

**Authors:** Maciej Borowiec, Dominika Lech, Robert Maksymowicz, Jeremi Matysek, Cyprian Strączek, Aleksandra Strzelecka, Marcin Kozakiewicz, Łukasz Krakowczyk, Krzysztof Dowgierd

**Affiliations:** 1 Head and Neck Surgery Clinic for Children and Young Adults, Regional Specialized Children’s Hospital in Olsztyn, 18a Żołnierska Str., 10-561 Olsztyn, Poland; 2 Department of Clinical Pediatrics, Head and Neck Surgery Clinic for Children and Young Adults, University of Warmia and Mazury, 10-709 Olsztyn, Poland; 3 Department of Maxillofacial Surgery, Medical Univeristy of Lodz, 113 Żeromskiego Str., 90-549 Lodz, Poland; 4 2nd Department of Oncologic Surgery, Maria Sklodowska Curie Memorial National Cancer Center, 44-100 Gliwice, Poland

**Keywords:** pediatric microsurgery, free flap reconstruction, head and neck surgery, donor-site morbidity, flap survival, functional outcomes

## Abstract

**Background:** Microsurgical free-flap reconstruction has become essential for restoring form and function in pediatric head and neck defects, yet outcome data remain heterogeneous and often limited to technical survival. This single-center retrospective study evaluated 54 free-flap procedures performed in 46 pediatric patients (16 months–18 years) to assess clinical effectiveness using a standardized Treatment Effectiveness Score integrating flap viability, donor-site healing, and functional recovery. **Methods:** Recipient- and donor-site outcomes, swallowing, mastication, breathing, and mouth opening were systematically assessed, and age was analyzed as a modifying variable. **Results:** Overall flap survival reached 76% and mean Treatment Effectiveness Score in cases without flap loss was 89.7. Partial complications occurred in 37% of procedures, while donor-site morbidity was rare (5.6%). Full functional restoration across all domains was achieved in 80.4% of patients, with most impairments occurring in combined osseous and soft-tissue reconstructions. Age demonstrated no significant association with either flap survival or overall treatment effectiveness. Continuous postoperative Doppler monitoring facilitated early identification of vascular compromise and supported salvage attempts in selected cases. **Conclusions:** These findings support the safety and feasibility of pediatric microsurgical reconstruction across the age spectrum and demonstrate that composite outcome scoring provides a more comprehensive assessment than flap survival alone, offering a framework for standardized reporting in future studies.

## 1. Introduction

Microsurgical reconstructions have transformed the management of complex defects across surgical disciplines [1,2]. The introduction of the operating microscope and reliable microvascular anastomosis enabled the routine transfer of free flaps for composite tissue reconstruction [1]. Within head and neck surgery, free-flap techniques are now indispensable for restoring form and function after oncologic resection, trauma, and correction of congenital anomalies [3,4]. Although the pediatric population benefits from the same conceptual advances, children present distinctive challenges—smaller vessel caliber, evolving craniofacial growth, and donor-site considerations that extend into adulthood—making pediatric outcomes a domain that cannot be extrapolated from adult experience alone [2,4,5].

Early pediatric applications of microsurgery included replantation and limb reconstruction, demonstrating the feasibility of microvascular repair in small vessels and young patients [1,2]. As experience accumulated, free flaps were increasingly applied to craniofacial indications, including osseous reconstruction of the mandible and midface (fibula, iliac crest, medial femoral condyle) and soft-tissue resurfacing with perforator flaps such as the anterolateral thigh [4,5,6]. Contemporary pediatric series consistently report high flap survival, often exceeding 90% [4,6,7]. Long-term follow-up of mandibular reconstruction with vascularized free fibula demonstrates not only reliable survival but also favorable symmetry and growth compatibility [7,8,9]. However, controversy remains regarding the role of patient age, with some authors identifying increased risk of failure in younger children, while others report no significant age effect [2,5]. Similarly, flap type and recipient site may influence outcomes, as maxillary reconstructions appear to carry higher failure rates than mandibular ones [10].

Despite encouraging results, the evidence base for pediatric head and neck free-flap reconstruction remains heterogeneous. Many publications aggregate diverse indications (oncology, trauma, congenital), flap types, and recipient sites across multiple institutions, while outcome reporting varies widely. Most series emphasize technical survival, whereas functional results and donor-site morbidity are underreported. Standardized composite scoring systems are rare, limiting comparability across studies and reducing the potential for evidence-based guidance [2,4,5,7].

To address these gaps, the present study evaluated the clinical effectiveness of pediatric head and neck free-flap reconstruction at a single specialized center using a pragmatic, internally developed composite outcome measure. We applied the Treatment Effectiveness Score, which integrates flap viability, recipient-site healing, donor-site morbidity, and clinically relevant functional outcomes assessed over postoperative follow-up. This score was designed to provide a structured summary of multidimensional reconstructive outcomes rather than a formally validated patient-reported instrument. By examining this composite endpoint alongside key clinical variables-such as patient age, flap type, and recipient site—we sought to describe patterns of reconstructive success in children and to explore the feasibility of composite outcome reporting in pediatric microsurgical reconstruction.

## 2. Materials and Methods

This was a single-center observational study based on retrospective analysis of medical records. All procedures were performed at the Department of Head and Neck Surgery, Regional Specialized Children’s Hospital in Olsztyn, Poland. The study covered the period from 2013 to 2018 and included pediatric patients aged 16 months to 18 years. A total of 46 children (22 boys, 24 girls) underwent 54 free flap procedures.

- 26 patients underwent tumor resection with simultaneous reconstruction.
- 20 patients underwent reconstruction only (18 due to craniofacial malformations, 1 due to chemical burn of the oral cavity, and 1 due to traumatic maxillary defect).
- 8 children required an additional flap procedure (2 for a subsequent tumor in a different location, 6 due to necrosis of the primary flap).

For statistical analysis, cases were categorized by diagnosis, recipient site, flap type and type of reconstruction (primary vs. secondary).

### 2.1. Outcome Measures

The Treatment Effectiveness Score was developed as a pragmatic composite outcome measure intended to capture the multidimensional success of pediatric head and neck free-flap reconstruction. It integrates flap viability, recipient-site healing, donor-site morbidity, and clinically relevant functional outcomes. The weighting of individual components was based on expert consensus within our multidisciplinary team and reflects their anticipated long-term clinical impact. Functional impairments were weighted more heavily than transient wound complications, as they are more likely to persist and affect quality of life. The score has not undergone formal external validation and should be considered an internally developed clinical assessment tool. This represents a limitation of the present study.

The primary outcome measure was the Treatment Effectiveness Score assessed at the last available follow-up. Secondary outcomes included flap survival, recipient- and donor-site complications, and individual functional domains. Functional assessment was performed no earlier than 6 months postoperatively to allow for wound maturation and adaptation. The minimum follow-up was 6 months.

#### 2.1.1. Treatment Effectiveness Score

Clinical effectiveness was evaluated using the Treatment Effectiveness Score (0–100 points).

- 100 points indicated proper integration of the flap at the recipient site and complete healing of the donor site.
- 0 points indicated total flap loss due to necrosis.

Point deductions were applied according to predefined criteria:

Recipient site: wound dehiscence (−10), marginal necrosis (−10), hematoma within 3 days (−10), infection within 30 days (or within 1 year in the presence of artificial materials; −10), major bone resorption at 6 months assessed by the modified Abyholm scale (−10).

Recipient site function: impairment of swallowing (−20), mastication (−20), breathing (−20), or mouth opening reduction compared with preoperative status (−20).

Donor site: wound dehiscence after 2 weeks (−10), infection within 30 days (−10), hematoma within 3 days (−10).

#### 2.1.2. Healing Assessment

Recipient site healing was defined as primary wound closure without complications. At day 7 postoperatively, wound inspection included assessment for dehiscence and marginal necrosis. Hematomas were confirmed by ultrasonography. Infections were diagnosed within 30 days (or up to 1 year in cases with prosthetic materials) by clinical examination and microbiological testing where indicated.

Donor site healing was assessed during follow-up visits for wound dehiscence, infection (microbiological swab), and hematoma (clinical and ultrasonographic confirmation).

#### 2.1.3. Functional Assessment

Functional outcomes at the recipient site were assessed using predefined clinical criteria adapted from published adult or mixed-population functional assessment literature, due to the lack of pediatric-specific validated instruments for microsurgical head and neck reconstruction.

Swallowing was evaluated by clinical observation of safe intake of liquids and soft foods without aspiration, coughing, or regurgitation, supplemented by caregiver-reported feeding difficulties [11,12].

Mastication was assessed based on patient- or caregiver-reported ability to chew age-appropriate solid food, supported by clinical evaluation of occlusion and mandibular mobility [13].

Breathing was evaluated clinically at rest and during mild exertion, with attention to signs of upper airway obstruction.

Mouth opening (trismus assessment) was measured by comparing preoperative and postoperative interincisal distance, using the four-finger test when indicated, and interpreted according to pediatric normative data [14].

Functional impairments and scar-related soft tissue abnormalities were incorporated into the Treatment Effectiveness Score using predefined point deductions, informed by published data on facial scar morphology and healing patterns [15].

### 2.2. Age as a Variable

Patient age at the time of surgery was included as an independent variable. Analyses were performed both with age as a continuous variable and by categorical grouping (≤5 years, 6–10 years, 11–15 years, ≥16 years). Associations between age and clinical outcomes were tested, including Treatment Effectiveness Score, flap survival, and the presence of complications at the recipient and donor sites.

### 2.3. Postoperative Flap Monitoring

During the first 7 postoperative days, flap viability was monitored using a modified Kruse et al. score sheet [16]. Parameters included handheld Doppler signal, flap color (physiologic/pale/cyanotic), and capillary refill of the skin island. Doppler monitoring was performed with a portable Bidop ES-100 VX, 8 MHz device (Hadeco, Poznań, Poland). Lack of Doppler signal, abnormal flap color, or absence of capillary refill prompted urgent evaluation for possible vascular compromise. Monitoring was conducted by the same investigator throughout the study period.

### 2.4. Data Collection and Statistical Analysis

Data were extracted from hospital records and standardized evaluation forms. Statistical analysis was performed using STATISTICA 13.3 PL. For comparisons of continuous variables across multiple groups, ANOVA or Kruskal–Wallis test was applied, depending on distribution. Relationships between categorical variables were assessed using the χ^2^ test of independence and correspondence analysis when appropriate. Spearman’s correlation was used for associations between continuous variables, including age and Treatment Effectiveness Score. The significance threshold was set at *p* < 0.05.

## 3. Results

### 3.1. Overall Treatment Effectiveness

A total of 54 microsurgical free-flap reconstructions were performed in 46 pediatric patients (22 boys and 24 girls). Donor-site healing was satisfactory in 41 of 54 reconstructions (75.9%). The mean Treatment Effectiveness Score for reconstructions where flap loss did not occur was 89.7, with 23 cases achieving 100 points.

**Figure 1:**
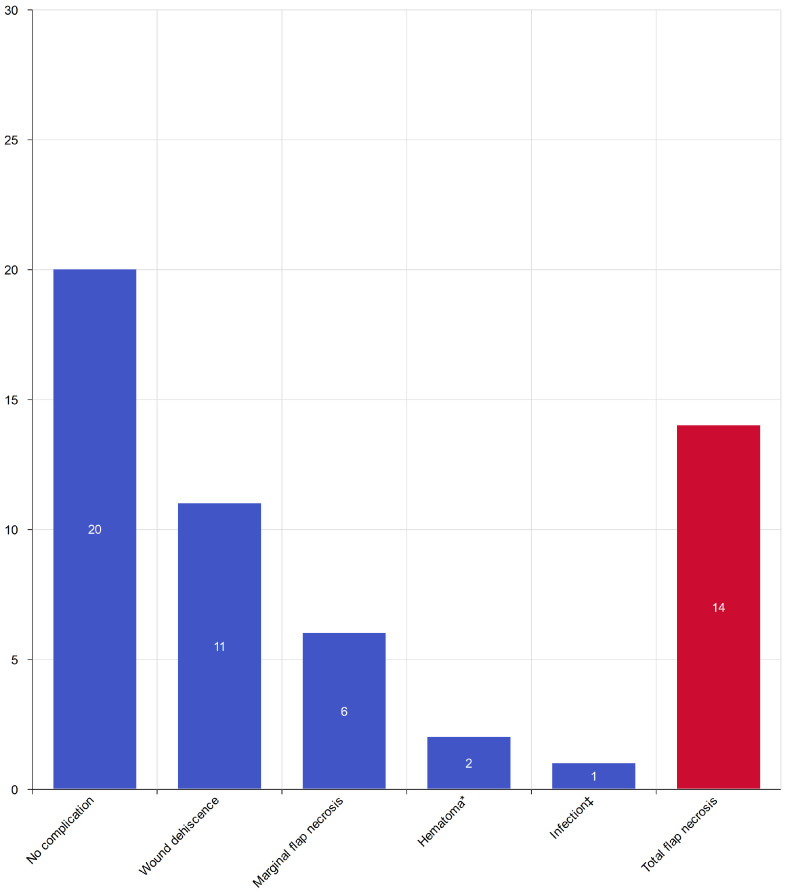
Distribution of postoperative recipient site complications after microsurgical free-flap reconstruction in the study cohort. Bars show the number of cases with no complication and with specific complications, including marginal flap necrosis, wound dehiscence, hematoma formation, infection, and total flap necrosis. (*—hematoma formation within 3 postoperative days; ‡—infection within 30 postoperative days).

Figure 1 indicates that the overall flap survival rate was 76%. Total flap necrosis occurred in 14 of 54 reconstructions (25.9%), all of which required secondary reconstructive procedures. Partial complications at the recipient site were observed in 20 of 54 reconstructions (37%).

- marginal necrosis—6 cases (11.1%),
- wound dehiscence—11 cases (20.3%),
- hematoma formation within 3 postoperative days—2 cases (3.7%),
- infection within 30 days—1 case (1.8%).

Primary reconstructions (after oncologic resection, n = 26) demonstrated a mean score of 88.2 ± 6.9, while secondary reconstructions (n = 20) achieved 86.9 ± 7.6; this difference was not statistically significant (*p* = 0.41). There was no significant difference in mean scores between reconstructions for tumor-related (n = 26) and congenital or traumatic deformities (n = 20) (*p* = 0.47).

### 3.2. Functional Outcomes

**Figure 2:**
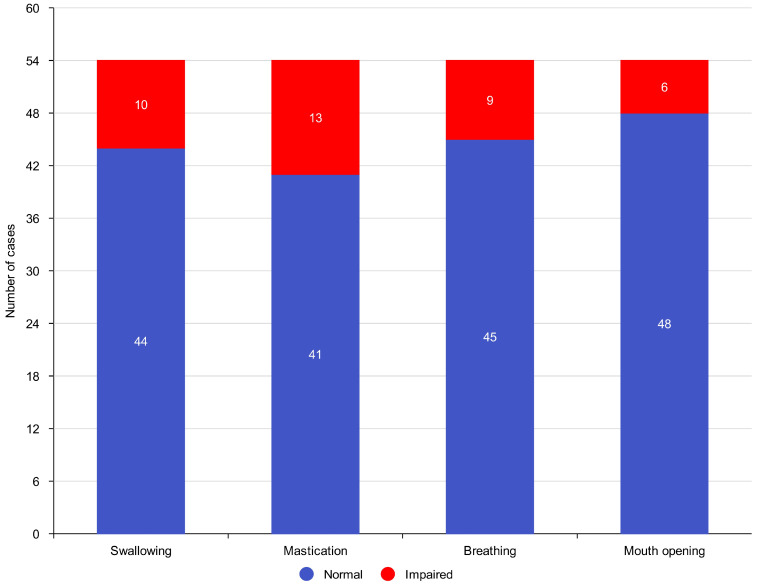
Functional outcomes at the recipient site after reconstruction in the study cohort. Bars show the number of cases classified as normal versus impaired for each functional domain: swallowing, mastication, breathing, and mouth opening. Impairment was defined according to the functional component of the Treatment Effectiveness Score assessment.

The median duration of postoperative follow-up was 12 months (range: 6–24 months). Outcomes were assessed at the last available follow-up visit, no earlier than six months postoperatively. Functional recovery was achieved in the majority of patients, which is presented in Figure 2.

- Normal swallowing was preserved in 44 patients (95.7%).
- Effective mastication was maintained in 41 patients (89.1%).
- Normal breathing function was observed in 45 patients (97.8%).
- Mouth opening reduction (trismus) occurred in 6 patients (13%).

Overall, 37 patients (80.4%) achieved full restoration of all four major functions (swallowing, mastication, breathing, mouth opening). Functional impairments were more frequent in patients after combined resection of bone and soft tissue, particularly in mandibular and maxillary reconstructions.

### 3.3. Flap Viability Monitoring

Postoperative flap viability was monitored using the modified Kruse et al. [16] score sheet during the early postoperative period. Signs of vascular pedicle compromise were detected in all 14 reconstructions that ultimately resulted in total flap necrosis. In each case, a complete absence of handheld Doppler signal was observed, accompanied by progressive changes in skin paddle coloration from a physiological appearance to cyanotic and subsequently pale or grayish discoloration. Capillary refill was absent on digital pressure in all affected flaps. Despite recognition of vascular compromise, irreversible thrombosis led to flap loss in these cases.

Continuous handheld Doppler monitoring (Bidop ES-100 VX, 8 MHz) proved reliable for early detection of vascular insufficiency, including in younger patients under 5 years of age.

### 3.4. Age-Dependent Differences

**Figure 3:**
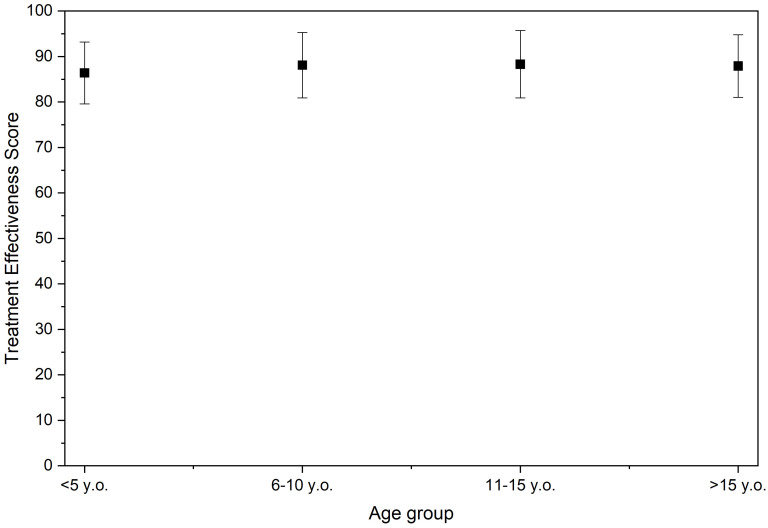
Dot plot with error bars representing mean and standard deviation of Treatment Effectiveness Scores for different age groups at the time of surgery. Points represent the mean score for each age category, and error bars represent the standard deviation. The *y*-axis ranges from 0 to 100, where higher values indicate better overall treatment effectiveness.

Figure 3 shows that when stratified by age group, the mean Treatment Effectiveness Scores were as follows:

- ≤5 years: 86.4 ± 6.8
- 6–10 years: 88.1 ± 7.2
- 11–15 years: 88.3 ± 7.4
- ≥16 years: 87.9 ± 6.9

**Figure 4:**
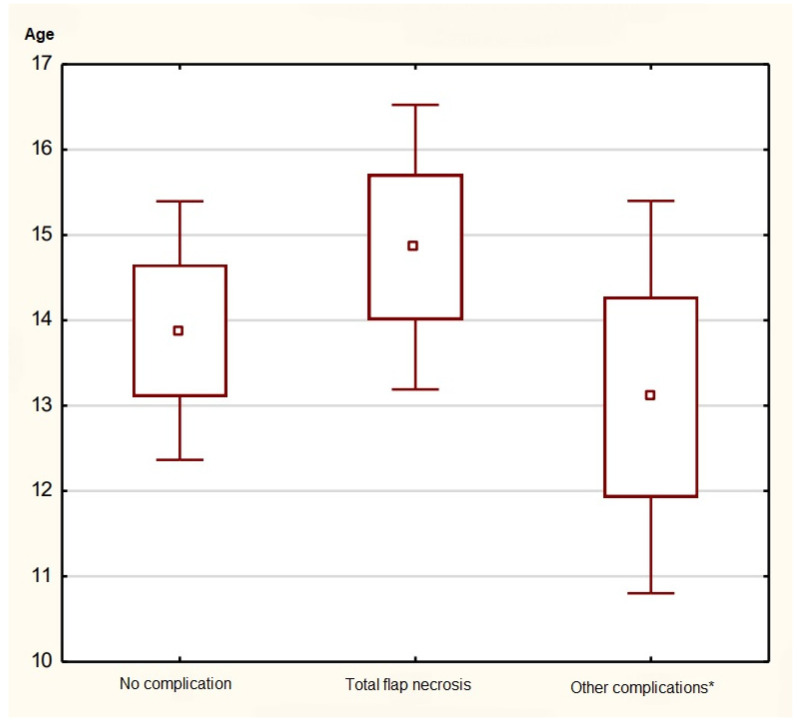
Plot representing mean age at the time of surgery according to types of complications (*—other complications include hematoma, partial necrosis, wound dehiscence). The plot compares age distributions between cases with no complication, total flap necrosis, and other complications.

As shown in Figure 4, no statistically significant differences were found among the age groups (ANOVA, *p* = 0.56). Minor complications (hematoma, partial necrosis, wound dehiscence) occurred slightly more often in the ≤5-year group (21.4%) than in older children (16.7%), though the difference was not significant. No association was found between patient age and flap loss (Spearman’s ρ = −0.12, *p* = 0.48).

## 4. Discussion

Reconstructive medicine and microsurgery have progressed rapidly, with microvascular free flaps becoming foundational in reconstruction after trauma, oncologic resection, and chronic wounds. Our analysis evaluates efficacy using a structured, point-based treatment-effectiveness and flap-monitoring chart. Standardized, continuous monitoring enables early recognition of compromise and prompt re-intervention, which is consistently associated with better outcomes [17,18,19,20]. Because tissue biology, comorbidities, and physiological reserve vary by age, perioperative strategies should be adjusted across the pediatric age spectrum [4,21].

The cohort was stratified by age, sex, procedure type, and indication. The treatment effectiveness score captured the full therapeutic arc—from recipient-site flap integration and functional performance to donor-site healing—rather than focusing solely on anastomotic patency. Although much of the literature prioritizes patency as the primary endpoint [22,23,24,25,26,27,28], extensive reconstructions depend on multiple perioperative and postoperative determinants. In cases with successful flap integration, the mean effectiveness score was 89.7, with 23 cases achieving the maximum of 100 points, supporting high effectiveness in children. However, the score should be interpreted as a pragmatic, internally developed clinician-assessed tool rather than a validated outcome instrument. In particular, it is not a patient-reported outcome measure, has not undergone external validation, and its semi-quantitative functional domains may be influenced by observer interpretation. Because comparable score-based studies are lacking, we interpreted each score component against existing evidence.

Recipient site assessment included delayed closure, culture-confirmed infection, and ultrasound-verified hematoma. We interpreted the outcomes while considering modifiers such as age, wound location, healing modality, intercurrent wound problems, and incision alignment with relaxed skin tension lines. Prior pediatric series report wound-healing complications in a minority of cases, most commonly partial dehiscence and less often infection or scar-related sequelae [29,30]. In our material, dehiscence occurred in 27% (11 procedures), 10 of which involved fibular flaps. Marginal skin-paddle necrosis occurred after six operations (14% overall), again predominantly with fibular bone flaps. Importantly, broader datasets underscore that clinically relevant complications are not limited to anastomotic failure and include hematoma, infection, and wound-healing disturbance [31].

Hematoma is particularly consequential. Pressure near the venous pedicle can impair outflow and precipitate flap loss. Large series, including pediatric cohorts, identify hematoma-related pedicle compression as a recurrent pathway to re-exploration and flap failure [27,30,32]. Despite the complexity of our cases, infections were infrequent, consistent with effective antibiotic prophylaxis aligned with institutional policy. Low infection rates in pediatric microsurgery have also been reported previously [32].

Postoperative swallowing, mastication, and respiration were preserved across the cohort. Importantly, no malignant tongue or floor-of-mouth tumors were included; thus, wide oncologic margins—often detrimental to function—were unnecessary. Swallowing, assessed by the modified water swallow test, remained intact in all patients [11], contrasting with the dysphagia commonly seen after adult oncologic resections involving the tongue/floor of mouth [12]. Masticatory function, tested using the van den Engel-Hoek protocol (6 min chewing of a logopedic bite block with VAS pain reporting), showed no deficits [11]. The Jorine A. Vermaire two-color wax mixing test, frequently used after head-and-neck resections, is unsuitable in children due to aspiration risk [13]. Mouth opening, evaluated by the three-finger Zawawi test, was generally normal [14]. Exceptions included two patients with Pruzansky III hemifacial microsomia reconstructed with fibular flaps who developed restricted opening related to overcorrection and soft tissue tension; absence of a glenoid fossa further complicated alignment. One patient reconstructed with an iliac crest flap developed an intraoral cheek hypertrophic scar limiting opening; similar restriction followed an anterolateral thigh (ALT) flap due to hyperplastic scarring. Consistent with these observations, a Korean series of 428 facial postoperative scars found hypertrophic scarring in 18.5% without obvious impediments to healing [15]. These findings argue for meticulous planning of mandibular ramus/TMJ reconstruction and long-term functional surveillance.

Donor-site evaluation used congruent criteria: non-primary intention healing, culture-confirmed infection, and ultrasound-verified hematoma. Dehiscence was most frequent after fibular harvest (three cases), uniformly in the distal third of the leg. Pediatric skin’s reduced elasticity increases closure tension and dehiscence risk; for larger skin paddles, split-thickness skin graft (STSG) coverage is prudent [33]. Early fibular donor-site complications (infection/dehiscence) have been reported at 9.9%, rising to 19% when STSG is employed. Other donor sites performed predictably: abdominal wall layers allow tension-free closure after iliac crest harvest; the radial forearm is routinely resurfaced with STSG with reliable healing; and the medial femoral condyle skin paddle is typically small enough for primary closure [34,35].

We observed two infections overall—one donor, one recipient—likely driven by exogenous factors, given the rarity of pediatric systemic risks (e.g., diabetes, cachexia). This underscores strict asepsis during long microsurgical cases and attentive postoperative care. Donor-site hematoma occurred in four patients (one iliac crest, three fibula). Ultrasound and re-exploration failed to reveal a discrete bleeding source, implicating generalized oozing from osteotomy lines and emphasizing meticulous bone-edge hemostasis. Donor-site hematoma is generally reported as infrequent in both pediatric and adult microsurgical series [31,36,37].

Overall free-flap survival in our cohort was 76%, which is lower than that reported in some contemporary pediatric reports: large series typically report flap survival above 90%, while adult head-and-neck series also commonly exceed 90% despite differences in case mix and prior radiotherapy exposure [22,30,32,38]. The lower survival rate observed in the present study therefore warrants careful and balanced interpretation. Several interrelated factors may have contributed. First, a substantial proportion of reconstructions were performed for congenital deformities and post-resection defects, in which recipient vessels may be hypoplastic, anatomically variable, or altered by prior surgery and scarring. Although facial vessels were most commonly used as recipient vessels, their variable anatomical course and caliber in pediatric patients occasionally necessitated the use of alternative recipient vessels, thereby increasing procedural complexity. Flap survival depends not only on intraoperative anastomotic patency but also on postoperative surveillance and timely recognition of vascular compromise. In pediatric patients, limited cooperation, difficulties with immobilization, and reliance on indirect clinical signs may affect early detection of flap compromise, underscoring the importance of structured monitoring protocols and caregiver education [30]. Finally, institutional experience, cumulative case volume, and the learning curve inherent to complex pediatric microsurgery should be acknowledged as relevant contextual factors. The observed survival rate should therefore be interpreted in light of case mix, anatomical complexity, and evolving perioperative and monitoring protocols rather than as an isolated measure of technical performance.

By flap type, success was highest with medial femoral condyle flaps. Failures clustered among osteocutaneous radial forearm flaps used for bilateral cleft maxillary reconstruction, where severe post-cleft scarring, mucosal deficiency, oronasal fistulae, limited vestibular space, need for a long pedicle, and the contaminated intraoral milieu complicate inset, surveillance, and healing. Notably, this clustering of failures suggests that the observed outcomes are more closely related to the specific requirements of these defects and the suitability of the selected flap than to isolated technical issues at the level of microvascular anastomosis. This interpretation is consistent with the finding that all flaps left the operating room with patent anastomoses and that failures were concentrated in a distinct reconstructive subgroup rather than being evenly distributed across flap types. Reports of free-flap solutions for similar defects are scarce; a small adult series reconstructed alveolar clefts with medial femoral condyle flaps without donor-site complications [39]. This pattern underscores the importance of defect-driven flap selection in complex pediatric maxillary reconstruction.

Pediatric vessels differ from adult vessels in caliber and reactivity, including a tendency toward vasospasm. Gilbert and Shapiro reported minimum safe anastomotic diameters of 0.7 mm and 0.5 mm, respectively; in our series, no vessels under 1 mm were encountered, easing anastomosis [40,41]. Evidence regarding heightened pediatric vasoreactivity is mixed. Operations in children under five are often technically more demanding, whereas outcomes at ≥10 years approximate adult results [42]. Vascular characteristics in adolescents (13–17 years) are reported to closely resemble those in adults [43]. Conversely, Liu observed a higher rate of failure in the 5–9-year group [32]. In our material, age did not significantly correlate with recipient-site complications, including necrosis (*p* = 0.5255).

This study has several limitations that should be considered when interpreting the findings. First, the Treatment Effectiveness Score is a pragmatic, internally developed composite measure and should not be interpreted as a validated outcome instrument. The score is not a patient-reported outcome measure and has not undergone external validation. Moreover, some components—particularly the semi-quantitative assessment of functional domains such as swallowing, mastication, breathing, and mouth opening—may be influenced by observer interpretation, which is especially relevant in pediatric patients due to variable cooperation and reliance on caregiver-reported information. Nevertheless, the components were selected to reflect clinically meaningful events and functions routinely assessed in postoperative follow-up (recipient-site healing, donor-site healing, and core head and neck functions). Reporting both the composite score and its individual components provides transparency and reduces the risk that the composite metric obscures clinically important patterns. Second, direct comparison of flap survival rates across studies should be interpreted with caution, as reported outcomes are influenced by multiple factors, including institutional experience, patient selection, case complexity, flap type distribution, and postoperative monitoring protocols. In pediatric patients in particular, postoperative management may be challenging because of limited cooperation, difficulties in immobilization, and the need to rely on indirect clinical signs during flap monitoring. Additionally, postoperative monitoring and most functional assessments were performed by a single investigator, which reduced inter-observer variability but may have introduced observer bias, particularly in the evaluation of semi-quantitative outcomes. Finally, the study cohort included patients reconstructed for oncologic, traumatic, and congenital indications, resulting in an etiologically heterogeneous population. In the present study, subgroup sample sizes were insufficient to permit formal etiology-specific outcome analyses, and such comparisons were therefore not undertaken. The decision to analyze the cohort as a pooled population was guided by prior research from our group in a substantially larger cohort, which demonstrated no significant differences in reconstructive outcomes attributable to etiology [7]. In the current cohort, flap types were distributed across indication groups without evident clustering, further supporting pooled analysis for the study objectives.

## 5. Conclusions

Pediatric microsurgical free-flap reconstruction is a safe and effective method for managing complex head and neck defects. In this single-center cohort, flap survival was lower than that reported in most contemporary pediatric series and donor-site morbidity was low, with most patients achieving satisfactory functional recovery. No statistically significant association between patient age and outcomes was detected, supporting the feasibility of microsurgery across the pediatric age spectrum, while acknowledging the limited statistical power of age-stratified analyses. Importantly, the flap-specific failure pattern observed in complex maxillary and cleft-related reconstructions highlights the clinical relevance of defect-driven flap selection in pediatric head and neck microsurgery. The Treatment Effectiveness Score was applied as an exploratory, pragmatic framework to integrate flap viability, recipient- and donor-site healing, and functional outcomes beyond anastomotic patency. As an internally developed, clinician-assessed composite measure, it should be interpreted cautiously and cannot be considered a validated outcome instrument. Nevertheless, the present findings suggest that composite outcome reporting may offer a useful complementary perspective in pediatric microsurgical reconstruction, warranting further validation and external application in larger, multicenter cohorts.

## Data Availability

The data are available upon request from the corresponding author.
